# Renal fibrosis detected by diffusion-weighted magnetic resonance imaging remains unchanged despite treatment in subjects with renovascular disease

**DOI:** 10.1038/s41598-020-73202-0

**Published:** 2020-10-01

**Authors:** Christopher M. Ferguson, Alfonso Eirin, Abdelrhman Abumoawad, Ahmed Saad, Kai Jiang, Ahmad F. Hedayat, Sanjay Misra, James Glockner, Stephen C. Textor, Lilach O. Lerman

**Affiliations:** 1 Division of Nephrology and Hypertension, Mayo Clinic, 200 First St SW, Rochester, MN 55905 USA; 2 Department of Radiology, Mayo Clinic, Rochester, MN USA

**Keywords:** Cardiology, Nephrology

## Abstract

Tissue fibrosis is an important index of renal disease progression. Diffusion-weighted magnetic resonance imaging’s (DWI-MRI) apparent diffusion coefficient (ADC) reveals water diffusion is unobstructed by microstructural alterations like fibrosis. We hypothesized that ADC may indicate renal injury and response to therapy in patients with renovascular disease (RVD). RVD patients were treated with medical therapy (MT) and percutaneous transluminal renal angioplasty (MT + PTRA) (n = 11, 3 bilaterally, n = 14 kidneys) or MT (n = 9). ADC and renal hypoxia (R2*) by blood-oxygen-level-dependent MRI were studied before (n = 27) and 3 months after (n = 20) treatment. Twelve patients underwent renal biopsies. Baseline ADC values were correlated with changes in eGFR, serum creatinine (SCr), systolic blood pressure (SBP), renal hypoxia, and renal vein levels of pro-inflammatory marker tumor necrosis-factor (TNF)-α. Renal oxygenation, eGFR, and SCr improved after MT + PTRA. ADC inversely correlated with the histological degree of renal fibrosis, but remained unchanged after MT or MT + PTRA. Basal ADC values correlated modestly with change in SBP, but not in renal hypoxia, TNF-α levels, or renal function. Lower ADC potentially reflects renal injury in RVD patients, but does not change in response to medical or interventional therapy over 3 months. Future studies need to pinpoint indices of kidney recovery potential.

## Introduction

Renovascular disease (RVD), such as renal artery stenosis, may lead to chronic kidney disease (CKD) and potentially end-stage renal failure^1^. Renal blood flow (RBF) is compromised by significant RVD, which also promotes hypertension, decreases glomerular filtration rate (GFR), and induced interstitial fibrosis in stenotic kidneys (STK)^1^. Treatment of RVD conventionally consists of anti-hypertensive medical therapy (MT), alone or in combination with percutaneous transluminal renal angioplasty (MT + PTRA), and stenting to restore RBF to the STK^2^. Renal functional recovery in response to therapy in patients with RVD is variable and may include further loss of kidney function^3^. However, the ability of restoration of RBF to alleviate fibrosis remains unknown.

Several imaging modalities and techniques have been deployed to assess renal microstructure. Diffusion-weighted imaging (DWI) magnetic resonance imaging (MRI) is a potent non-invasive tool for assessment of alterations in tissue microstructure^4–6^, particularly attributed to fibrosis. DWI does not require the use of exogenous contrast agents, which is especially important in patients with impaired renal function^7^. Apparent diffusion coefficient (ADC) is a quantitative index derived from DWI by fitting diffusion-weighted images to the respective MR signal using a mono-exponential decay model^8^. ADC is simple to implement and is achievable by as few as two different sets of diffusion-weighted images in renal pathological conditions^9^. Low ADC values, reflecting impediment to water diffusion, are considered to indicate changes in microstructure, such as tubular atrophy or interstitial fibrosis^8^.

The severity of tissue damage is an important determinant of renal outcomes in RVD^10^. DWI has been previously shown to have potential for noninvasive assessment of renal fibrosis in CKD^11^. STK ADC falls in patients with RVD^12^, and inversely correlates with STK fibrosis in swine RVD^8^, but whether ADC is altered in response to treatment in patients with RVD remains unknown. We hypothesize that DWI-ADC values would correlate with renal damage and with renal response to medical therapy, but would remain unchanged after treatment with PTRA.

## Results

All MT + PTRA patients underwent successful PTRA and stenting, three of which were bilateral. SBP in the MT + PTRA patient group fell significantly after the procedure, as did mean arterial pressure, but remained unchanged after MT (Table 1). At baseline MT + PTRA patients were taking a larger number of lipid-lowering and antihypertensive medications. Basal eGFR and SCr was similar in the treatment groups, but in MT + PTRA eGFR rose and SCr fell at 3 months, and renal BOLD-MRI R2* declined. Contrarily, renal function and oxygenation remained unchanged in the MT group. There was no difference or change in TNF-α in either.

**Table 1:** Systemic and renal characteristics before and 3 months after treatment in patients with RVD treated with medical therapy without or with percutaneous transluminal renal angioplasty.

Parameter	Medical therapy		Medical therapy + revascularization	
	Baseline	Follow-up	Baseline	Follow-up
**Demographics**				
Number of patients/kidneys	9/9	9/9	11/14	11/14
Age (year)	69.6 ± 7.7	69.6 ± 7.7	–	–
Sex (female/male)	6/3	*–*	4/7	–
Body mass index (kg/m^2^)	27.6 ± 4.7	27.2 ± 4.7	28.8 ± 3.8	29.3 ± 4.6
**Blood pressure (mmHg)**				
Systolic	133.8 ± 18.0	129.4 ± 12.7	151.2 ± 17.3#	137.7 ± 13.0*
Diastolic	65.2 ± 7.7	64.0 ± 7.0	70.5 ± 11.5	65.1 ± 9.9
Mean	88.1 ± 10.1	85.8 ± 7.6	97.4 ± 11.3	89.3 ± 9.7*
**Concomitant medications**				
No of antihypertensive drugs	2.0 ± 1.4	2.0 ± 1.4	3.3 ± 1.3#	3.1 ± 1.6
Lipid-lowering drugs (no of patients)	9/9	8/9	7/11#	7/11
**Renal function and injury**				
eGFR-CKD-EPI (mL/min/1.73/m^2^)	54.0 ± 17.7	46.3 ± 10.8	45.6 ± 19.6	54.4 ± 18.4*
Serum creatinine (mg/dL)	1.3 ± 0.4	1.3 ± 0.4	1.6 ± 0.4	1.3 ± 0.3*
STK-BOLD-MRI R^2^* (1/s)	22.2 ± 3.7	22.0 ± 4.3	24.9 ± 5.0	21.9 ± 3.8*
STK-TNF-α (pg/ml)	16.3 ± 12.4	14.6 ± 8.3	11.2 ± 5.9	11.4 ± 5.7

*p ≤ 0.05 versus baseline and #*p* ≤ 0.05 vs. medical therapy. eGFR (CKD-EPI): estimated glomerular filtration rate (chronic kidney disease epidemiology collaboration), BOLD-MRI: blood oxygen-level-dependent magnetic resonance imaging, TNF-α: tumor necrosis factor-α, STK: stenotic kidney.

**Figure 1:**
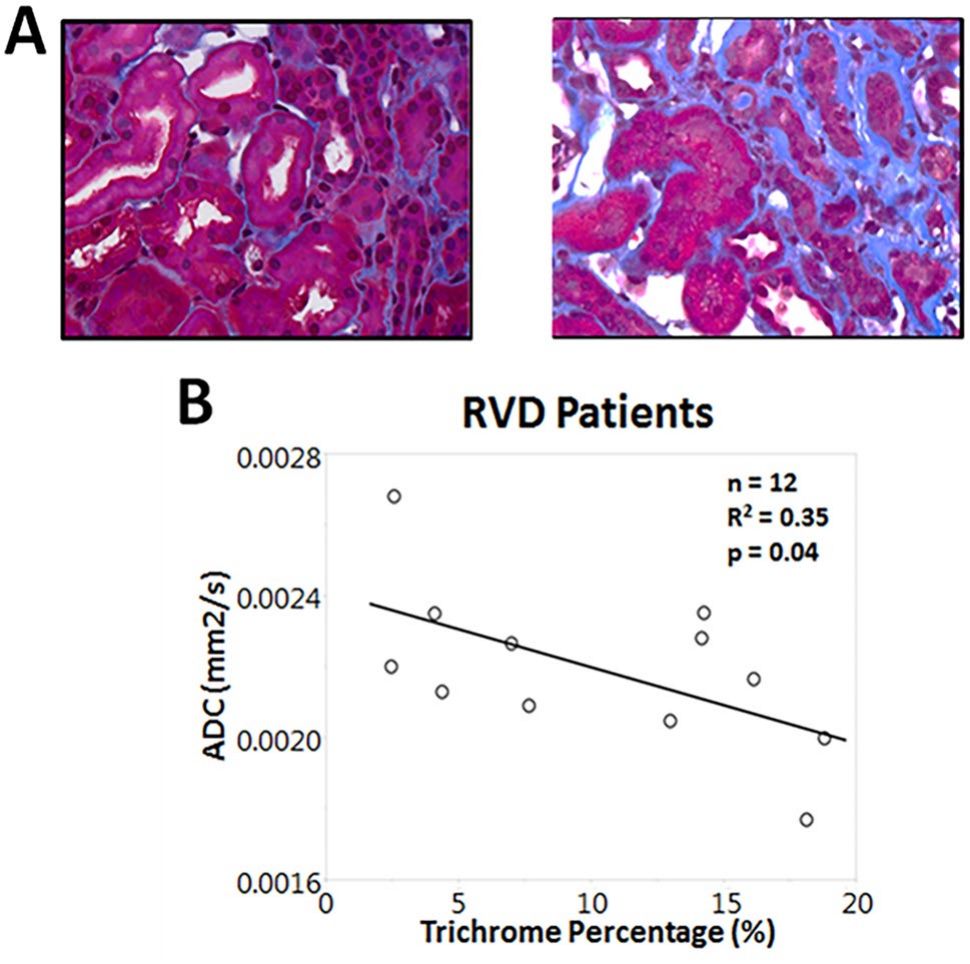
(**A**) Representative histology images from two different stenotic kidney biopsies stained with Mason’s trichrome. The image on the right illustrates a higher degree of renal fibrosis. (**B**) Inverse correlation between percentage of trichrome-stained stenotic kidneys and ADC values in RVD patients (R^2^ = 0.35, *p* = 0.04).

**Figure 2:**
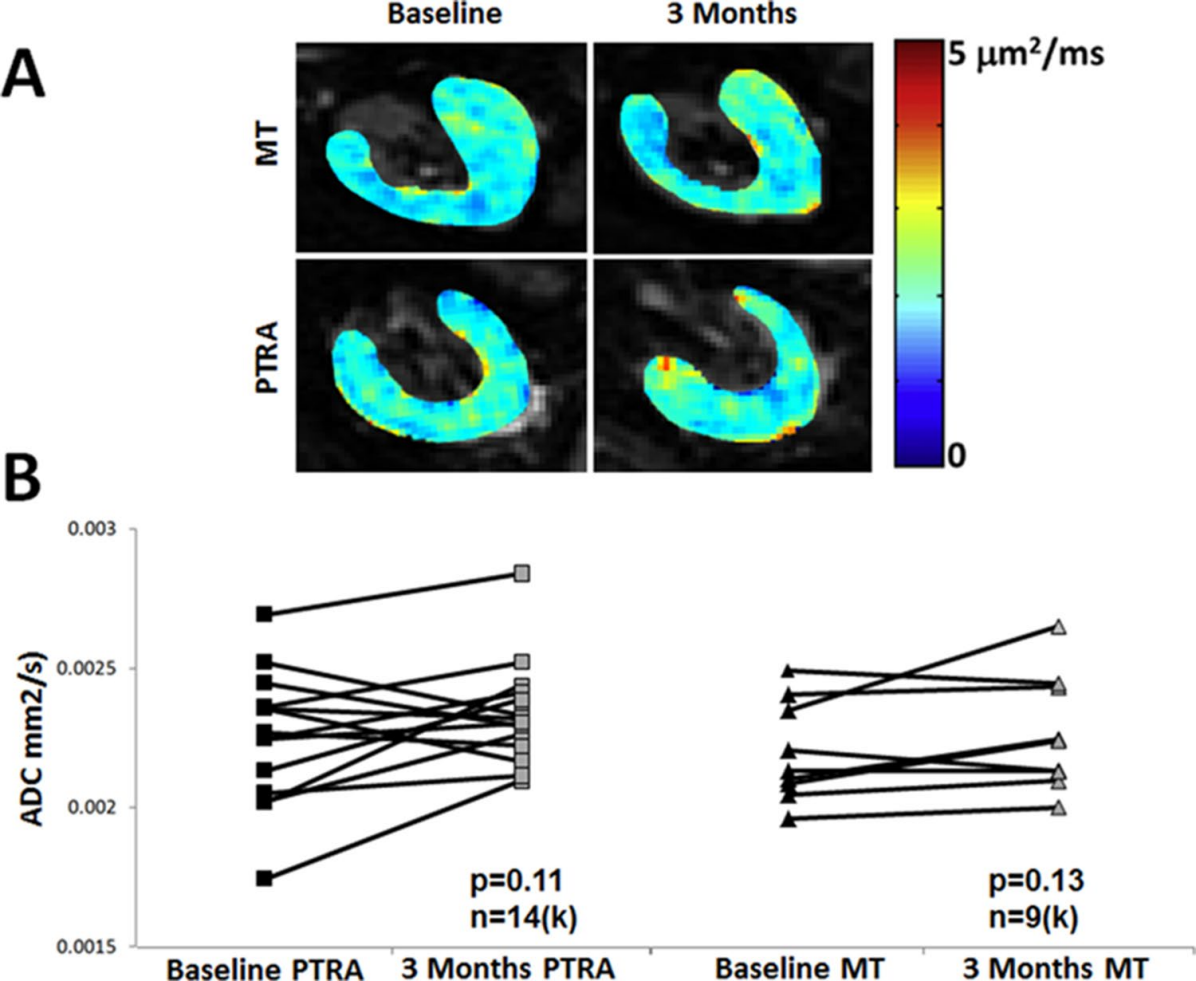
(**A**) Representative DWI-ADC maps depicting similar stenotic-kidney ADC value distributions at baseline versus post-treatment in MT and MT + PTRA treated patients. The bar on the right shows the ADC scale. (**B**) ADC values before compared to 3 months after treatment in kidneys (K) of MT + PTRA (*p* = 0.11) and MT (*p* = 0.13) treatment groups.

STKs biopsied at baseline (n = 12) showed mild fibrosis (Fig. 1A, 10.2 ± 5.9%) by trichrome staining, which inversely correlated with basal ADC values (Fig. 1B, R2  = 0.35, *p* = 0.04), highlighting the potential of DWI to detect kidney damage non-invasively. However, ADC values remained unchanged at follow-up in both MT + PTRA (*p* = 0.11) and MT (*p* = 0.13) (Fig. 2A,B).

**Figure 3:**
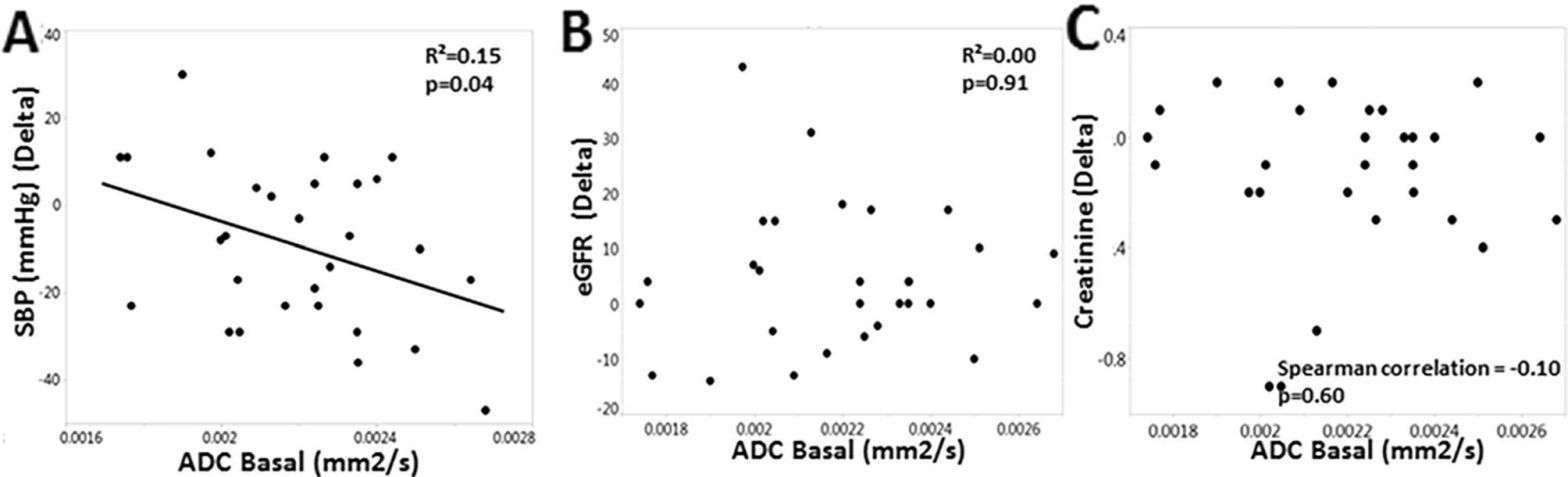
(**A**) Inverse correlations of basal ADC with the change in systolic blood pressure (R^2^ = 0.15, *p* = 0.04). Basal ADC did not significantly correlate with the change (delta) in either (**B**) eGFR (R^2^ = 0.00, *p* = 0.91) or (**C**) serum creatinine (spearman correlation = − 0.10, *p* = 0.60) over the 3-months treatment period.

Basal ADC values in all RVD patients did not correlate with either BOLD R2* or TNF-α. Basal ADC values showed a moderate correlation with the change in delta SBP (R^2^ = 0.15, *p* = 0.04) although it did not correlate with the levels or change in eGFR or SCr (Fig. 3). Basal trichrome staining and BOLD-MRI R2* did not correlate with these functional parameters either (both *p* > 0.05).

## Discussion

This study shows that ADC values may reflect kidney injury, and remain unchanged in response to revascularization in RVD patients. ADC values in RVD patients were correlated inversely with renal fibrosis suggesting a link to kidney damage. Furthermore, RVD patients undergoing PTRA, but not those treated only medically, showed an improvement in renal function. Nevertheless, ADC values did not correlate with renal function or response to therapy in either group. These observations suggest that renal fibrosis remains unchanged 3 months after renal revascularization, regardless of functional outcomes.

RVD may be identified in up to 7% of adults over 65 years of age in the United States, and may lead to loss of renal function and development of renovascular hypertension. Alas, recent clinical trials have shown that restoration of renal arterial patency with revascularization does not necessarily lead to improvement of renal function, although there is some small benefit for decreasing systolic BP^3^. Contrarily, renal revascularization seems to be effective in selective cohorts of patients with RVD, such as those with low levels of albuminuria^13^. Renal fibrosis is also considered to be an important biomarker, determinant of renal outcomes, and a therapeutic target in CKD^9^, which can predict outcomes in renal transplant recipients and native kidney disease.

The advent of MRI has yielded effective techniques to identify renal fibrosis such as DWI MRI, a powerful technique sensitive to changes in microstructure of tissues^8,14^. With initiatives such as PARENCHIMA, collection of highly technical data such as DWI and uniform parameters for successful acquisition are more readily available^5,15^. The DWI index, ADC, is considered to be a marker of interstitial fibrosis or tubular atrophy in the kidney. For example, in kidney allograph recipients, ADC inversely correlates with fibrosis, and BOLD imaging, indicative of oxygen availability^16^, also correlates with moderate-to-severe interstitial fibrosis, particularly when > 50%^17,18^. However, the ability of DWI to correlate with renal outcomes in RVD has not been determined. Our study confirms that in a subgroup of patients with RVD, ADC values correlate with interstitial fibrosis determined by trichrome staining, underscoring its ability to detect changes in renal microstructure.

In addition, we compared ADC before and 3 months after treatment in patients undergoing MT alone or MT + PTRA. We found that in neither group did ADC change after therapy. We also observed a significant rise of eGFR and fall in SCr in patients undergoing revascularization, but not in those continuing on MT, although the ultimate levels achieved were not significantly different between them. BP, which was initially elevated in MT + PTRA relative to MT patients, declined to comparable levels in patients treated with MT + PTRA, demonstrating the potential of revascularization to improve BP control and renal function in selected patients.

Nevertheless, ADC levels remained unchanged in both groups, and did not correlate with eGFR, SCr, renal hypoxia or inflammation. These observations imply that renal damage in RVD is determined by factors other than measurable tissue fibrosis alone. Interestingly, these observations suggest that ADC is an adequate index of renal injury, but not a suitable predictor of renal response to therapy.

## Limitations

Given the complex nature and careful uniformity of our inpatient protocol, our sample size was relatively small. In addition, the b-values were collected during separate acquisitions, which may lead to some motion or misregistration artifacts. Newer DWI methods allow for acquisition of multiple b-values during a single acquisition period^9^. However, we excluded most b-values less than 300 s/mm^2^ to minimize the effects of renal perfusion on ADC. Our timeframe to assess regression of tissue fibrosis (3 months) may have been too short to determine conclusively if only renal function improves rather than fibrosis. Further studies will need to include a larger number of patients followed for a longer period of time post-treatment.

## Conclusions

Taken together, our results demonstrate that ADC values correlate inversely with renal levels of fibrosis, underscoring its utility to detect renal injury. On the other hand, ADC remained unchanged after MT or MT + PTRA, and did not correlate with either eGFR or SCr, indicating that renal microstructure assessed by ADC does not change in response to therapy in patients with RVD and mild tissue fibrosis. Larger patient studies at longer duration of follow-up are needed to determine the ability of this or other tools to identify patients that might benefit from specific treatment modalities.

## Methods

### Patients

The study was approved by the institutional review board and was compliant with the Accountability and Health Insurance Portability Act. Each patient provided written and informed consent. Twenty-seven patients with RVD over the age of 18 years were prospectively recruited. Renal artery stenosis was determined by Doppler velocities values ≥ 300 cm/s. Exclusion criteria included renal disease necessitating dialysis, serum creatinine (SCr) > 2.5 mg/dL, major medical conditions (angina, cancer or stroke), a non-functioning kidney, contradictions for MRI, and/or technical issues at the baseline or follow up. Seven patients were excluded from follow-up due to technical issues related to MRI scans.

The patients underwent an inpatient protocol consisting of 3 days in the Mayo Clinic clinical research trials unit with a regulated dietary constraint of sodium intake (150 mEq/day) and an isocaloric diet. Blood oxygen-level dependent (BOLD-MR) imaging studies were performed on day 2, and tumor necrosis factor (TNF-α) levels (Luminex, Millipore Burlington, MA) measured in renal vein samples collected during computed tomography (CT) studies on day 3 for an unrelated protocol^19,20^. Estimated GFR (eGFR) was calculated by CKD epidemiology collaboration (CKD-EPI).

All patients were treated with angiotensin receptor blockers (ARB) or angiotensin converting enzyme inhibitors (ACE), but loop diuretics were temporarily held so as not to interfere with BOLD-MRI. Doses of ACE and ARB varied between patients, were prescribed based upon clinical decision to achieve blood pressure control and were consistent between visits and throughout the duration of the study. Patients then continued either treatment with (MT, n = 13) alone or with additional renal revascularization (MT + PTRA, 3 bilaterally, n = 17 kidneys) based on clinical decision. Only DWI studies that used identical protocols (b-values) at baseline and follow-up were included, resulting in 9 MT and 14 (3 bilaterally) MT + PTRA kidneys in the final analysis. Prior to stenting, the right STKs of twelve RVD patients were transvenously biopsied by way of the jugular vein, as previously described^19^. All RVD patients returned after 3 months and all measurements repeated with the exception of biopsy.

Laboratory and clinical parameters included age, sex, body mass index (BMI), systolic (SBP), diastolic (DBP), and mean blood pressure (BP), SCr, TNF-α, eGFR, and cholesterol levels were evaluated at baseline and after 3 months of treatment by standard protocols^21^.

### MRI study

#### BOLD

BOLD MRI was performed as described^22^ using a 3 T (GE Medical Systems, Milwaukee, WI) scanner with a Fast Gradient Echo with multiple echo times. MR axial images were acquired during breath-hold (≤ 20 s). BOLD-MRI mostly followed PARENCHIMA guidelines, and the MRI parameters used in this study as well as PARENCHIMA's recommendations are summarized in the Supplementary Table 1^16,23^.

For data analysis, an in-house MatLab (The MathWorks, Natick, MA) graphical user interface was used to quantify BOLD data. Regions of interest (ROIs) drawn on T2* images were subsequently transferred to R2* BOLD maps. In each kidney slice where BOLD images were generated, a large ROI was drawn ecompassing the total kidney (cortex and medulla). Total renal oxygenation was quantified by averaging all R2* values within in each slice of the kidney^20,24^.

#### DWI

DWI was acquired as previously described^25^ using the 3T scanner with a total of 3–8 axial images collected. DWI-MRI was mostly performed in accordance with PARENCHIMA guidelines. All of the parameters used in this study are described and compared to PARENCHIMA's recommendations in Supplementary Table 2^5,15^.

To analyze DWI data^8^, renal ADC maps were created by pixel-by-pixel fitting the MR signal intensity versus b-values using a mono-exponential decay model. ROIs were traced encompassing the kidney in each b_0_ DWI image, generating ADC maps in each slice. ADC maps were sampled using single large ROIs that covered the entire kidney (cortex and medulla). The mean values for ADC in each ROI was calculated and then averaged across all slices of the kidney. We have shown that both ADC and intra-voxel incoherent motion parameters correlated well with STK fibrosis^8^.

#### Morphological studies

Renal fibrosis was quantified from trichrome-stained renal sections as described^8,26^. In brief, 10–15 images were acquired for each slide at 20× using a microscope (ZEN 2012 blue edition Carl ZEISS SMT, Oberkochen, Germany). The percentage of fibrotic (blue) tissue was assessed using a semi-automatic approach^19^.

#### Statistical analysis

Statistical analysis was performed using JMP (v.14 pro, SAS Institute, Cary, NC). Data that was normally distributed was expressed as mean ± SD and compared using either a paired or unpaired two-tail t-test, as appropriate. Data that was non-normally distributed was compared using a non-parametric test (Wilcoxon or Wilcoxon signed rank). Correlations were tested with Pearson correlation (normally-distributed) or Spearman’s correlation (non-parametric); *p* ≤ 0.05 was considered statistically significant.

### Ethics approval and consent to participate

The study adheres to the principles of the Declaration of Helsinki and all human subjects have given their informed consent. Approval of this study was granted by the Mayo Clinic Institutional Review Board (IRB).

## Supplementary information

Supplementary Tables.

## Data Availability

All data generated or analyzed during this study are included in this published article.

## Abbreviations

RVD: Renovascular disease
CKD: Chronic kidney disease
RBF: Renal blood flow
GFR: Glomerular filtration rate
STK: Stenotic kidney
DWI: Diffusion-weighted imaging
MRI: Magnetic resonance imaging
ADC: Apparent diffusion coefficient
SCr: Serum creatinine
BOLD: Blood oxygen-level dependent
TNF-α: Tumor necrosis factor
CT: Computed tomography
eGFR: Estimated GFR
CKD-EPI CKD: Epidemiology collaboration
MT: Medical therapy
MT+PTRA: Medical therapy plus percutaneous transluminal renal angioplasty
BMI: Body mass index
SBP: Systolic blood pressure
DBP: Diastolic blood pressure
BP: Blood pressure
ROI: Regions of interest
