# Supplementary material for: Renal fibrosis detected by diffusion-weighted magnetic resonance imaging remains unchanged despite treatment in subjects with renovascular disease
Source: Sci Rep. 2020 Oct 1;10:16300. doi: 10.1038/s41598-020-73202-0 (PMC7530710; doi:10.1038/s41598-020-73202-0)
Supplement: Supplementary file 1 — Supplementary Tables. [file 41598_2020_73202_MOESM1_ESM.docx]

**Supplemental Material**

**Renal fibrosis detected by diffusion-weighted magnetic resonance imaging remains unchanged despite treatment in subjects with renovascular disease**

**Running head:** Diffusion-weighted magnetic resonance imaging in renovascular disease

Christopher M. Ferguson¹, Alfonso Eirin¹, Abdelrhman Abumoawad^1^, Ahmed Saad^1^, Kai Jiang^1^, Ahmad F. Hedayat^1^, Sanjay Misra², James Glockner², Stephen C. Textor¹, Lilach O. Lerman¹

**Supplementary Table 1.** Parameters used for acquisition of DWI-MRI: the current study compared to PARENCHIMA recommendations.

|  | DWI | | |
| --- | --- | --- | --- |
| Parameter | | PARENCHIMA recommendation | Study parameter/ conformity |
| Preparation | | Normal hydration | ✔ |
| Field Strength | | 1.5 T or 3.0 T | ✔,3.0 T |
| Sequence | | Single shot echo planar imaging | ✔ |
| Orientation | | Oblique coronal | Axial |
| Matrix | | >128 | ≥128 |
| In-plane resolution | | 2-3 mm | ✔,2.3 mm |
| Slice thickness | | >4 mm | ✔,7 mm |
| Coverage | | Full kidney | ✔ |
| Parallel imaging factor | | 2 | ✔ |
| Fat suppression | | SPAIR | ✘ |
| TR (s) | | 4 s | 2-2.4 s |
| TE (ms) | | Min (<100) | ✔, 60- 94 ms |
| Averages | | 3 | 2 |
| Breathing mode | | Respiratory gated (or free breathing with post-hoc motion correction) | ✔, Manual Breath-hold |
| Cardiac gating | | no | ✔ |
| Diffusion gradients | | Monopolar | ✔ |
| ADC # b-values, # directions, b-values | | 4, 3, 0,100,200,800 s/mm^2^ | ✔4,3, 0,300,600,900 s/mm^2^ |
| Time (min) | | 2 | <2 |
| Distortion correction | | Recommended | ✔ |
| Registration | | Recommended, unilateral if possible | ✔ |
| Image quality control | | Recommended | ✔ |
| ROI placement | | b=0 image | ✔ |
| Cortical ROI | | 1 stripe / slice:>3 slices | ✘ |
| Medullary ROI | | 3 samples / slice:>3 slices | ✘ |
| Reporting | | Cortex and Medulla | Entire kidney, multiple ROIs |
| Metrics statistics reporting | | Mean, Median, Standard deviation, ROI size | Mean, Standard deviation, Large ROI |
| Diffusion units | | 10^-3^ mm^2^ / s | ✔ |
| Map format | | Colormap, fused with anatomy if possible | ✔ |

DWI-MRI: diffusion weighed imaging- magnetic resonance imaging, ROI: region of interest, SPAIR: Spectral attenuation recovery

**Supplementary Table 2.** Parameters used for acquisition of BOLD-MRI: the current study compared to PARENCHIMA recommendations.

|  | | BOLD | |
| --- | --- | --- | --- |
| Parameter | PARENCHIMA recommendation | | Study parameter used/ conformity |
| Preparation | Normal hydration (100 ml water), 4 h fasting from food | | ✔ |
| Field Strength (B_0_) | 1.5 T or 3.0 T (preferred) | | ✔,3.0 T |
| Sequence | 2D mGRE | | ✔,2D fast multi-gradient echo |
| Orientation | Coronal oblique to kidneys | | Axial |
| In-plane resolution | 2-3 mm | | 1.1 x 1.7mm |
| Slice thickness | 3-5 mm | | ✔, 5 mm |
| Coverage | 3-5 slices centered on renal hilum | | ✔ |
| Parallel imaging factor | 2 | | ✔ |
| Fat suppression | Yes | | Yes |
| TR (s) | 60-75 ms | | >75 ms |
| TE (ms) | 8-16 echoes, ≤50 ms (~T2 cortex) at 3T; choice of in phase for fat-water | | ✔,Less than 50 ms (w/ minimum of 12 echo times) |
| Averages | 1 | | ✔ |
| Breathing mode | Breath hold | | ✔ |
| Image quality control | Recommended | | ✔ |
| ROI placement | Manual | | ✔ |
| Cortical ROI | 1 stripe/slice; > 3 slices | | ✘ |
| Medullary ROI | 3 samples/slice; > 3 slices | | ✘ |
| Fitting | Mono-exponential or log-linear | | ✔, Mono-exponential |
| Reporting | Cortex and medulla | | Entire kidney |
| Reported metric | R_2_* (s^-1^) | | ✔ |
| Metric statistics reporting | Mean, median, standard deviation, ROI size | | Mean, Standard deviation, Large ROI |
| Map format | Color or grayscale quantitative map | | ✔ |

BOLD-MRI: blood oxygen-level-dependent blood magnetic resonance imaging, ROI: region of interest, 2D mGRE: two-dimensional multiple gradient echo
